# Corporate political activity in the context of sugar-sweetened beverage tax policy in the WHO European Region

**DOI:** 10.1093/eurpub/ckac117

**Published:** 2022-09-13

**Authors:** Kathrin Lauber, Holly Rippin, Kremlin Wickramasinghe, Anna B Gilmore

**Affiliations:** Department for Health, University of Bath, Bath, UK; WHO European Office for the Prevention and Control of Non-Communicable Diseases (NCD Office), Division of Country Health Programmes, WHO Regional Office for Europe, Moscow, Russia; WHO European Office for the Prevention and Control of Non-Communicable Diseases (NCD Office), Division of Country Health Programmes, WHO Regional Office for Europe, Moscow, Russia; Department for Health, University of Bath, Bath, UK

## Abstract

**Background:**

Sugar-sweetened beverage (SSB) taxes have emerged as an effective and increasingly popular tool to reduce added sugar intake, an important contributor to obesity and non-communicable diseases. A common barrier to the implementation of well-designed SSB taxes is the opposition of commercial actors. Focusing on the WHO European Region, this study seeks to map if and how key stakeholders have experienced industry efforts to influence SSB taxes.

**Methods:**

We identified 11 countries in the WHO European Region which have implemented SSB taxes or attempted to do so. Using an online survey informed by the global literature on industry interference with SSB taxation, we approached 70 in-country policymakers, advocates and academics. The data were analysed using an existing framework of corporate political activity.

**Results:**

Twenty-three experts from nine countries responded to the survey. Transnational SSB producers and their business associations were identified as the most active opponents of SSB taxation. Industry claims that the policy would have negative economic effects were identified as the most common and powerful arguments. Direct lobbying was reported in all study countries. Shifts in political activity were recognisable across stages of the policy process, moving from outright opposition to attempts to delay or weaken the policy after its announcement.

**Conclusion:**

Those seeking to introduce effective SSB taxation can use our findings to pre-empt and counter industry opposition. We identify several measures for preventing and mitigating industry interference with SSB tax policy.

## Introduction

Unhealthy diets are a major contributor to non-communicable diseases (NCDs) and mortality worldwide.^1^ Although efforts to address these dietary risks to date have predominantly focused on voluntary measures, nutrient-poor and energy-dense sugar-sweetened beverages (SSBs) have emerged as a target for statutory regulation. Growing evidence indicates that taxing SSBs works to reduce consumption^2^ and the World Health Organization (WHO) recommends a 20% *ad valorem* tax as a cost-effective means towards reducing NCDs.^1^ To date, over 40 countries globally have implemented SSB taxes, 10 of which are located within the WHO European Region.^3^

SSB tax designs vary widely, but most are excise taxes levied on industry rather than consumers. While many SSB taxes have been framed as public health measures, revenue generation features prominently as a primary motivation. Although a reduction in the intake of taxed products is a key health objective, tiered SSB taxes which apply differential tax rates based on sugar content have also successfully encouraged reformulation.^3^ Tax designs are not necessarily fixed; several countries such as France and Portugal have continued to amend their SSB taxes.^4^^,^^5^

Research indicates that public support for SSB taxation is good and increases with earmarking of tax revenue for health purposes and clear communication of the rationale.^6^ Yet, evidence suggests that commercial actors involved in the production and sale of SSBs have consistently opposed health taxes.^7–9^ An internal ‘public policy risk matrix’ produced by Government Relations managers for Coca-Cola Europe maps 49 regulatory policy threats to the company’s business, identifying SSB taxes as a priority lobby target with the recommendation to ‘fight back’.^10^ This threat is reflected in evidence of broader industry efforts aimed at blocking, delaying or weakening SSB taxes across the world, including at the European Union (EU) level.^11–18^

Although 10 countries in the European Region currently tax SSBs, others, such as Slovenia and Estonia, have withdrawn plans to introduce SSB taxes, and Norway recently abolished a long-standing SSB tax^3^ (see table 1). There remains a lack of in-depth, systematic research on the ways in which industry interference substantiates at the national level in the Region. Thus, we sought to explore if and how professionals involved in SSB tax processes in European Region countries have experienced industry interference. Specifically, we set out to address the following research questions:

**Table 1:** SSB taxation in the WHO European Region

Country	Tax design	Tax base	Exemptions	Announced	Implemented	Changes since implementation
Belgium	*Current:* Flat rate; €0.06/l	SSBs and ASBs (concentrated and RTD)	Milk products and replacements, fruit and vegetable juices	2015	2016	Raised multiple times since introduction.
	*First iteration:* Flat rate; €0.03/l					
Estonia	Tiered; increasing from €0.10/l (ASBs or 5–8 g sugar/100 ml) to €0.30/l (>8 g sugar/100 ml) *[not implemented]*	SSBs	Milk products, fruit and vegetable juices	June 2017	Not implemented	n/a
Finland^a^	*Current:* €0.32/l (>5% sugar), €0.13/l (other non-alcoholic beverages)	SSBs and ASBs (concentrated and RTD), juices, waters	Producers with an annual production volume of >50 000 l	Not identified	2011	Amended multiple times and last raised in 2020. A tax on certain high-sugar foods was withdrawn in 2017.
	*First iteration:* €0.22/l (sugar-containing soft drinks), €0.12/l (other non-alcoholic beverages)					
France	*Current:* Sliding scale; starting at ≥1 g sugar (and ASBs)/100 ml increasing up to €0.20/l for drinks >11 g sugar/100 ml	SSBs and ASBs	Milk products and replacements, special dietary foods	August 2011	January 2012	Amended in 2018 from a flat rate on soft drinks to sliding scale based on sugar content.
	*First iteration:* €0.716/l for ASBs and SSBs					
Hungary^a^	*Current:* Flat rate; HUF7/l on soft drinks, HUF200/l on syrups	SSBs and ASBs (concentrated and RTD)	Milk products, fruit and vegetable juices	July 2011	2011	Updated multiple times
	*First iteration:* HUF5/l on soft drinks					
Kazakhstan	To be determined	To be confirmed	To be confirmed	Not yet implemented	Not yet implemented	n/a
Latvia	€7.4/hl (<8 g/100 ml); €14/hl (≥8 g/100 ml) litre	SSBs and ASBs	Fruit and vegetable juices	Not identified	2004	Rate increased in 2016; amended in 2020 from flat rate to tiered design.
Norway^a^	NOK3.34/l *[abolished in 2021]*	SSBs and ASBs (concentrated and RTD)	Milk products, fruit and vegetable juices, products in powder form	Not identified	1924, updated 1981	Increased multiple times, last in 2018; abolished in July 2021.
Portugal	Tiered; increasing from €0.08/l (<80 g sugar/l) up to €0.16/l (for drinks with ≥80 g sugar/l)	SSBs and ASBs (concentrated and RTD)	Milk products and replacements, fruit and vegetable juices, special dietary foods	October 2016	February 2017	Amended from two to four tiers in 2019.
Republic of Ireland	Tiered, €16.26/hl on drinks with ≥5 g sugar/100 ml, €24.39/hl on drinks with ≥8 g sugar/100 ml	SSBs (RTD and concentrated)	Milk products and replacements, food supplements, alcohol replacements	October 2016	May 2018	Scope extended in 2019 to include certain plant protein drinks and drinks containing milk fats.
			Small-scale producers exempt from EU food labelling regulations			
UK	Tiered, £0.18/l (5–8 g sugar/100 ml), £0.24/l (>8 g sugar/100 ml)	SSBs (concentrated and RTD)	Milk products and replacements, alcohol replacements, fruit and vegetable juices, powdered drinks, special dietary foods.	March 2016	April 2018	n/a
			Small manufacturers which produce > 1 million litres of liable drinks per year			

Sources: Refs.^3^^,^^40^; see Supplementary appendix S2 for detailed sources.

ASB, artificially sweetened beverage; RTD, ready to drink; SSB, sugar-sweetened beverage.

a The policies of these countries also include(d) food products which are not discussed in detail here.

1. Which key arguments and practices have advocates, policymakers and researchers observed in countries in the Region that have introduced SSB taxes or sought to do so?
  - Which arguments and practices were seen as most prominent and impactful?
2. What may work to counter political activity in opposition to SSB taxation in the Region?

## Methods

We conducted a survey with key informants involved in policy processes around SSB taxation in the European Region. The survey was developed based on a literature review and refined through expert feedback. Ethical approval was obtained from the University of Bath Research Ethics Approval Committee for Health (REACH EP 20/21 059).

### Survey development

A scoping review of the empirical literature on corporate political activity (CPA) in the context of dietary public health policy (Supplementary appendix S1) was used to identify articles which presented empirical evidence on CPA around national-level SSB taxation. Papers which did not solely examine industry opposition to SSB taxes but presented relevant evidence, for instance, as part of a wider political economy analysis, were included. Using Atlas.ti,^19^ the lead author coded each instance where a political action or argument in the context of SSB taxation was mentioned in the Results section. We adopted the distinction between actions and arguments from the overarching structure of the Policy Dystopia Model, an evidence-based conceptualisation of CPA that is based on decades of tobacco industry research and has been successfully applied in obesity policy settings.^12^^,^^20^^,^^21^ Based on this analysis of the empirical literature, we developed a questionnaire to identify industry practices and arguments, contextual factors such as timing and actors, impacts and potential ways to address CPA. The draft questionnaire was tested by five academic experts and refined based on their feedback.

### Study country selection

We included all WHO Europe Member States which, as of December 2020, had successfully implemented an SSB tax, identified from the 2017 and 2019 WHO Country Capacity Surveys, the WHO Global nutrition policy review 2016–17, and informal expert input. Belgium, Finland, France, Hungary, Ireland, Latvia, Monaco, Norway, Portugal and the UK were initially identified as countries with taxes on SSBs (Poland implemented a tax in 2021, after the cut-off). Norway abolished its SSB tax shortly before the study period but was included in the study. We excluded Monaco as its SSB tax was introduced via an administrative process under a tax agreement with France. In addition, we included Estonia, where an SSB tax was adopted in 2017 but not implemented, and Kazakhstan, where an SSB tax was under development during the study period. This resulted in 11 included countries. All were excise taxes and covered SSBs, although some also included other beverages such as artificially sweetened beverages, juice and sweetened milk (see table 1). In some countries, taxes also applied to food products—this is out of scope for the current study and thus not discussed in detail here.

### Participant recruitment

We set out to recruit three to six participants from each country, comprising policymakers, advocates and academics/researchers who had been closely involved in, or had closely observed, the process towards the introduction of an SSB tax. Relevant individuals were identified via purposive sampling through WHO country focal points, the authors’ networks and snowball sampling. We invited participants via email to complete the online questionnaire and sent up to three follow-up messages over the 7-week study period (July to September 2021). We received 23 survey responses from nine countries, after sending 70 invites to 11 countries (participant characteristics in table 2). We received no responses from Finland and Latvia.

**Table 2:** Participant characteristics by country

Country	Participants				
	Policymakers	Civil society members	Academics/researchers	Other	Total
Belgium	1	–	–	–	1
Estonia	1	–	1	–	2
Finland	–	–	–	–	0
France	–	–	2	–	2
Hungary	2	–	–	–	2
Ireland	1	2	–	2	5
Latvia	–	–	–	–	0
Norway	1	3	1	–	5
Portugal	1	–	1	–	2
Kazakhstan	1	–	1	–	2
UK	–	1	1	–	2

### Analysis

Survey data were analysed qualitatively using Atlas.ti.^19^ Each survey response was coded by the first author using the instrumental strategies of the Policy Dystopia Model^22^ and the arguments identified via the literature review, with modifications where appropriate. Additional materials provided by participants and targeted web searches were used to triangulate survey information which related to specific events or activities. Where such specific incidents (rather than general observations) were concerned, companies and organisations are named only where we were able to support survey response data with a publicly available document. Based on this analysis, a brief synthesis was produced for each country. To validate our findings, initial syntheses were sent to all participants for feedback; at the same time, we approached one participant per country for a more in-depth follow-up call (based on the country synthesis). This resulted in written feedback from five participants and calls with four separate participants, insights from which were incorporated into country syntheses.

## Results

The vast majority of participants reported that they had observed interference from commercial actors around SSB tax policy. Business associations representing SSB producers or the wider food industry, and large SSB producers themselves were identified as the most active opponents in all countries (Supplementary appendix S3). The Coca-Cola Company (including local subsidiaries, Coca-Cola hereafter), for instance, was mentioned by 14 of 15 respondents who provided examples of companies opposing an SSB tax.

### Industry practices

Data indicated that industry actors engaged in four instrumental strategies from the Policy Dystopia Model^22^—direct involvement and access in policymaking, coalition management, information management and legal. Direct access and lobbying, media advocacy and pan-industry collaboration and coordination were the most frequently reported practices (table 3). Of these, lobbying of policymakers, either directly by industry actors or through professional lobbyists, was seen as the most impactful.

**Table 3:** Frequency of key practices, as reported by participants

Practice	Reported frequency					
	Never	Rarely	Sometimes	Frequently	All the time	I don’t know
Instrumental strategy: direct involvement and access						
Direct access/lobbying	–	–	2	11	7	3
Political donations	1	–	2	–	–	20
Intimidation/harassment	3	2	6	2	–	19
Instrumental strategy: coalition management						
Using seemingly independent third parties	2	3	7	4	2	5
Setting up anti-tax campaigns/groups	2	1	6	2	1	11
Collaborating within the industry	–	–	5	4	8	6
Instrumental strategy: information management						
Using evidence to oppose SSB tax	1	3	8	4	4	3
Media advocacy	2	–	6	9	3	3
Instrumental strategy: legal						
Legal action or threats	7	2	4	2	1	7

Numbers indicate the number of respondents who selected each option.

#### Coalition management

Business associations—either food- and/or beverage-specific or representing businesses from a country or region—were consistently highlighted as key to CPA. Transnational SSB producers were involved in most of the business associations mentioned by participants; Coca-Cola, for instance, was represented by of 11 of 13 named business associations which had published a membership list (Supplementary appendix S4). Conversely, one participant and an academic paper^4^ noted that in France, the ‘disorganised’ response of SSB companies may have undermined their efforts against the tax.

The use of broader coalitions beyond the food and beverage industry varied across countries—think tanks such as the Institute of Economic Affairs, for instance, were seen as particularly active in the UK, with some affiliated individuals and materials also appearing in the Irish SSB tax debate. In Ireland, one academic with industry ties was a vocal opponent of the tax in the public debate. Although no physical threats were reported, public health actors in several countries were subject to reputational challenges and intimidation, primarily in the form of media campaigns aimed at discrediting them.

#### Information management

Commercial actors commonly funded or produced their own research which challenged the need for SSB taxation or highlighted potential negative economic impacts. These outputs were mostly private reports rather than peer-reviewed publications. Food Drink Ireland, for instance, collaborated with analytics firm Creme Global to evaluate Irish food and beverage companies’ reformulation efforts,^23^ concluding SSB taxation was unnecessary.^24^

Alongside the production of industry-favourable evidence, industry actors have engaged in activities to disseminate information; when the Estonian SSB tax was under development, for example, the Estonian Food Industry Association hosted an international event on health taxes, with speakers from major European business associations UNESDA and FoodDrinkEurope.^25^ Moreover, media campaigns which promoted industry messaging were reported in the UK, Norway, France, Portugal, Ireland and Estonia.

#### Direct involvement and access

Direct involvement in, and access to, the policy process was reported in each study country, with most respondents (18 of 23) indicating that they observed this practice *frequently* or *all the time*. It manifested primarily as lobbying of decision-makers and participation in formal policy processes such as consultations. Ministries of Health and Finance were primary lobby targets, solicited by companies, their business associations or lobbying firms. This is illustrated in the case of the French SSB tax where Coca-Cola and Orangina-Schweppes lobbied officials in the lead-up to a planned amendment, not only directly but also through business associations such as ANIA and lobbying firms such as APCO and Rivington.^26^^,^^27^ An Estonian food industry actor reportedly hired the public relations firm Meta Advisory to lobby against SSB taxation; the latter’s website notes that its successes include ‘avoiding […] excise duties on soft drinks’.^28^ In the Irish case, Coca-Cola’s lobbying efforts included a meeting with the Taoiseach at the 2018 World Economic Forum.^29^

Government interaction with industry via multi-stakeholder platforms, forums for voluntary engagement between interested parties from public, private and other societal spheres, also provided key routes for access. In Norway, for instance, the business association FoodDrinkNorway participated in a committee tasked in 2018 by the Ministry of Finance with reviewing the country’s taxes on sugary products.^30^ Another route of direct pressure on decision-makers, closely linked to arguments around the economic harms of SSB taxes (see below), is what one participant termed ‘economic blackmail’; Coca-Cola, for instance, allegedly threatened to cancel planned investments in response to SSB tax proposals in France and Portugal.^31^^,^^32^ No concrete examples of SSB industry donations to politicians or political parties were reported.

#### Legal strategies

In several cases, industry actors claimed that SSB taxes contravened national or international legal obligations. In the Estonian case, for instance, industry assertions that the proposed SSB tax would be unconstitutional were highlighted as a potential factor in the President’s subsequent decision to veto the implementation of the policy. Legal challenges were most commonly threatened on grounds that SSB taxes would constitute ‘State aid’ for untaxed products or producers, in contravention of EU law. In the UK, for instance, the British Soft Drinks Association argued that the exemption for small producers may disadvantage large manufacturers by providing illegal aid to the former.^33^ The threshold for this exemption was subsequently lowered. Although complaints to the European Commission were reported regarding the Hungarian tax and a planned increase in the Norwegian tax,^34^ no formal cases have proceeded against SSB taxes in the study countries to our knowledge.

### Industry arguments

In their discursive strategies, industry actors employed several key arguments to highlight or exaggerate potential negative effects of SSB taxation and minimise potential health benefits (table 4). Most commonly, participants observed warnings of potential negative economic effects—either on the overall economy, employment or businesses themselves (often with emphasis on smaller businesses)—and claims that the policy was unfair or unjustified. This narrative was also perceived as most impactful by participants. Where SSB taxes were predominantly framed as a budgetary rather than a health measure during policy development (e.g., in France and Belgium), commercial actors opposed the policies on this basis, arguing that they were not designed and thus not suitable for protecting public health.

**Table 4:** Key industry arguments as reported by in-country informants and whether they are substantiated by independent evidence

Argument			Reported in the following countries								Supported by independent evidence?	Details
		FR	EE	IE	UK	PT	HU	NO	KZ	BE		
**Discursive strategy: expanding negative effects of SSB taxation**												
SSB taxation will harm the overall economy/cost jobs		✓	✓	✓	✓	✓	✓	✓	✓	✓	No	Job losses in SSB-related sectors tend to be offset by job creation in others. The same applies to macroeconomic concerns. Moreover, this argument draws on an artificial trade-off between health and the economy.
SSB taxation will harm businesses	Big businesses	✓	✓	–	–	–	✓	✓	✓	–	No	No peer-reviewed, independent study has shown that SSB taxes harm employment or the food sector. Taxation is intended to reduce the consumption of target products. However, existing evidence is insufficient to determine causal a relationship between SSB taxes and sector growth or changes to employment. The impact on share values appears minimal.
	Small businesses	✓	✓	–	–	–	✓	✓	✓	–	No	
SSB taxation poses an excessive administrative burden		–	✓	✓	–	✓	✓	✓	–	–	Uncertain	Fiscal measures do present administrative costs to companies, but well-designed taxes can minimise this.
SSB taxation will lead to illicit or cross-border SSB trade		–	✓	✓	✓	–	✓	✓	–	–	Uncertain	Although cross-border trade has been observed in some settings, it is primarily a concern for local jurisdictions and the overall evidence is mixed.
SSB taxation is unfair/discriminatory towards industry		✓	✓	✓	✓	✓	✓	✓	–	✓	n/a	Predominantly value-based/legal matter.
SSB taxation will disproportionately affect poorer people		✓	✓	✓	✓	✓	✓	✓	✓	–	Partially	Food and beverage taxes do present proportionally higher costs to low-income groups as these tend to spend a higher proportion of their disposable income on food compared with high-income groups. However, lower-income groups are also likely to benefit most from the health impacts of SSB taxes.^.^
SSB taxation impedes on people's freedom of choice/government is overstepping (‘nanny state’)		–	✓	✓	✓	✓	–	✓	–	–	n/a	This is predominantly value based so there is no evidence as such.
SSB taxation is a first step towards excessive regulation of other products ('slippery slope')		✓	✓	✓	✓	✓	–	✓	–	–	n/a	This is predominantly value based so there is no evidence as such.
Government is acting improperly (i.e. bad intentions, bad process)		✓	✓	✓	–	–	✓	✓	✓	–	n/a	This is predominantly value based so there is no evidence as such.
**Discursive strategy: questioning public health benefits of SSB taxation**												
SSB taxation will not work (i.e. not reduce consumption or obesity/NCDs)		✓	✓	✓	✓	✓	✓	✓	✓	–	No	Health taxes have an established economic rationale and three systematic reviews suggest that SSB taxes (>10–20% at point of purchase) effectively reduce SSB consumption. Although SSB taxes in isolation are not enough to ‘solve’ the obesity problem, studies modelling the potential long-term impact on obesity rates predict significant declines.
SSBs/sugar are not the problem (other products/behaviours are)		✓	✓	✓	–	✓	✓	✓	✓	–	No	Excess consumption of sugar, in particular SSBs, is a major cause of obesity, diabetes and cardiovascular disease.
SSB taxation is not evidence-based		✓	✓	✓	✓	–	✓	✓	✓	–	No*	See above. While a lot of the real-world evidence on the effects of SSB taxation has emerged in recent years, a significant amount of evidence linking SSB consumption to negative health outcomes, and modelling studies on the impacts of SSB taxation have been available for longer.
Industry is already addressing obesity/NCDs through voluntary efforts		✓	✓	✓	✓	✓	✓	✓	–	–	No	Voluntary sugar reformulation efforts have not been as successful as regulatory efforts. Reformulation prompted by tiered SSB taxes, on the other hand, has significantly reduced sugar content in SSBs. Meaningful voluntary action and tools such as education should be seen as complementary to taxation
SSB taxation will have negative public health consequences		✓	✓	✓	✓	✓	✓	✓	–	–	No	While poorly designed taxes do present a risk of prompting switching to less healthy products, well-designed SSB taxes are unlikely to have this effect.

Value-based arguments which cannot be addressed using evidence alone are denoted as such. Detailed sources can be found in Supplementary appendix S2.

### Temporal patterns

Industry political objectives and practices appeared to shift temporally. We illustrate this here using a simplified heuristic of policymaking in cyclical ‘stages’: agenda-setting, policy development, implementation and evaluation.^35^ Objectives of commercial opponents of SSB taxation were described by some respondents as shifting throughout the policy process, moving from outright opposition towards attempts to weaken an SSB tax or delay its introduction, to declaring the policy ineffective post-implementation. The use of coalitions such as business associations and less transparent allies, as well as attempts to undermine the credibility of public health actors, and direct lobbying were reported as occurring throughout policy processes but were most noticeable during the policy development stage (or when proposals to revise existing taxes were discussed). Media campaigns and dissemination of alternative evidence were prominent throughout agenda-setting and policy development stages; in the former case predominantly aimed at positioning voluntary approaches as a preferable solution, and in the latter case aimed at questioning the intended policy effect and warning of negative consequences. In Hungary, campaigns aimed at denying policy effectiveness around the time of the publication of official evaluation results were noted. Legal action or threats were reported primarily during policy development, including the revision of existing SSB taxes.

### What may work to prevent or mitigate industry interference?

Participants highlighted several ways in which industry interference could be addressed or mitigated. First, technical support from organisations such as the WHO, as well as targeted evidence were highlighted as key to robust, well-designed policies. Participants specifically noted that resources to pre-empt concerns and arguments raised by industry would be helpful. Second, a clearly communicated public health rationale and framing may help garner public support and avoid inadvertently feeding into opposing narratives. Similarly, fostering a broad public health presence in the public debate emerged as a potential way to mitigate the strong presence of industry positions in the media. This should focus on conveying the evidence and rationale underlying SSB taxation; additionally, the ‘polluter pays’ principle was highlighted as a potentially useful framing tool. Several respondents suggested that public health actors should also challenge the strong involvement of commercial actors in policy development given their clear conflicts of interest, while others remained favourable towards partnership working. Third, a strong, unified coalition which includes but is not limited to health was highlighted as important to countering well-organised anti-tax efforts. Lastly, the need for effective transparency measures surrounding political and charitable donations, lobbying and research funding was emphasised repeatedly.

## Discussion

We mapped industry involvement in SSB tax policy in nine WHO European Region countries and confirmed that the measures encounter consistent opposition from commercial actors who benefit from the sale of SSBs. Coca-Cola emerged as the most active opponent across countries, with local and regional business associations also playing a key role. SSB taxation touches on the commercial interests of a heterogenous set of actors within which disagreements may arise. While our data suggest that transnational corporations and their business associations consistently oppose SSB taxes at the outset, consensus can dwindle when it comes to policy design, with companies and their representative groups calling for their own products to be exempt while others are taxed.^15^^,^^36^ It is important to consider these findings in the wider context of evidence that transnational food industry actors have fought SSB taxes globally across levels of governance, including at the EU.^18^

Although the impact of industry activities cannot be stated with certainty, respondents generally considered that strong pushback contributed to weaker policy designs or delayed implementation. Participants in Estonia, which had abandoned plans to introduce an SSB tax in the final stages, and Norway, which had recently abolished a long-standing SSB tax, assigned industry a significant role in the outcome. Several factors were listed as potentially useful for preventing or countering industry interference, including a robust public health coalition with a unified position and strong media presence, technical preparedness and greater transparency surrounding lobbying in general.

A key issue raised by industry was inconsistency with trade law, in particular the concept of ‘State aid’ which, by distorting or threatening to distort competition, conflicts with EU law to the extent that it affects trade between states.^37^ However, well-designed SSB taxes are not necessarily incompatible with EU law. An assessment by the European Commission of Ireland’s SSB tax, requested by the Irish government, concluded that the tax on products with an added sugar content exceeding a set threshold ‘does not involve State aid’.^38^ Nonetheless, it is critical to assess the compatibility of planned SSB taxes with existing laws to strengthen a government’s position in the event of legal challenge and identify ways in which broader legal frameworks may facilitate the adoption of a tax. Several food taxes have, however, been ruled unlawful under EU law. Finland, for instance, abolished a confectionery tax following a European Commission ruling that the policy constituted unlawful State aid.^39^ While this does not mean that food taxes are generally incompatible with EU law, it highlights the importance of careful policy design—the key issue in both cases was exemption provisions which were interpreted to represent aid for non-exempt products.

Alongside the importance of cohesive and vocal public health coalitions, international cooperation and technical support, more general measures to increase policymaking transparency were highlighted as steps towards preventing or addressing industry interference in the context of SSB taxation. Most countries have either no or inadequate lobbying registers; even in the French and Irish registers that allow the public to see which policy areas were discussed in lobby meetings, information on amounts spent and detailed content of discussions is absent. Furthermore, our findings highlight the importance of independent policy evaluation: evidence from similar policies is crucial to counter industry arguments, whereas the lack thereof leaves a vacuum which tends to be filled with industry-commissioned reports. However, in doing so we must move away from narrow conceptions of acceptable evidence which make it significantly easier to demonstrate potential economic costs than long-term health benefits and allow complexity arguments to be invoked in favour of inaction. Lastly, the findings presented in this paper, alongside the wider literature, enable the pre-emption of industry opposition; using this as a basis to sensitise decision-makers early in the policy process may present a possible way forward to prevent industry influence and should be evaluated.

Overall, our findings also suggest that CPA in opposition to SSB taxes broadly aligns with the practices and arguments used by tobacco companies to undermine tobacco control measures.^8^ This indicates that learnings from tobacco control, including tools to address corporate interference in policymaking, are highly relevant to the public health community working to support the uptake of SSB taxes.

### Limitations

This research is exploratory in nature and subject to some methodological limitations. First, our findings are based on a relatively small number of respondents, as the initial pool of eligible individuals was limited. Second, this research only covers political activities which are observable and were observed—this may inadvertently exclude less overt activities. Similarly, participants may not have been positioned to disclose everything they remember due to the sensitive nature of the topic. Innovative methodological approaches could be explored to address some of these shortfalls, for example, to trace the impacts of lobbying or quantify elements of CPA. Lastly, we did not investigate countries where SSB taxation was not a concrete policy option. Our findings and a study from the Australian context, however, do suggest that commercial actors do engage in activities aimed at preventing SSB taxation from entering the policy agenda.^11^ As such, we recommend that this should be included in future research.

## Conclusion

Despite a now substantial body of supportive evidence, industry opposition against SSB taxes remains strong. Recent cases in which SSB taxes were overturned or not implemented highlight the immense task the public health community faces in introducing and maintaining effective public health regulations. In mapping how commercial opposition to SSB taxes has manifested, we present a first step towards building capacity to prevent and counter interference to support the implementation and protection of SSB taxes in the Region and beyond. To foster a shift to healthier food systems more broadly, further efforts are needed towards rectifying the power imbalance between those representing public health and commercial interests.

## Supplementary data

Supplementary data are available at *EURPUB* online.

## Supplementary Material

ckac117_Supplementary_Data

## Data Availability

The survey data underlying this article cannot be shared publicly due to the risk that participants may be identifiable. All additional information which was identified to support the survey data are in the public domain and linked as references.
