# Supplementary material for: Corporate political activity in the context of sugar-sweetened beverage tax policy in the WHO European Region
Source: Eur J Public Health. 2022 Sep 13;32(5):786–93. doi: 10.1093/eurpub/ckac117 (PMC9527967; doi:10.1093/eurpub/ckac117)
Supplement: ckac117_Supplementary_Data [file ckac117_supplementary_data.zip › Appendix 1.docx]

**Appendix 1:** Literature review

Scopus, Web of Science Core Collection, and PubMed were searched in April 2021 using terms related to products *(food, soda, beverage, drink*)*, regulatory areas *(advertis*/ marketing/ label*/ tax/ levy/ school food/ guidelines & policy/ legislat*/ regulat* & obesity/ non-communicable disease*/ public health)*, and corporate political activity *(political activit*/ lobb*/ interfere*/ influenc*/ argument* & industry*/ company*/ corporat*)* to identify relevant, peer-reviewed articles based on empirical research and published in English between 2010 and 2021*.*

All items included in the wider review were screened to establish if they include findings on industry interference with SSB taxation at the national level. In total, 25 relevant papers were identified, 15 of which specifically focused on SSB tax policy and 10 of which examined corporate political activity in general but included specific information in the context of SSB tax policy.

Content relevant to SSB taxation in each paper was coded in [Atlas.ti](https://atlasti.com/). The lower-level categories of practices and arguments were identified inductively and then classified into the overarching instrumental and discursive strategies from the [Policy Dystopia Model](https://doi.org/10.1371/journal.pmed.1002125), which fit well.
