# Supplementary material for: Corporate political activity in the context of sugar-sweetened beverage tax policy in the WHO European Region
Source: Eur J Public Health. 2022 Sep 13;32(5):786–93. doi: 10.1093/eurpub/ckac117 (PMC9527967; doi:10.1093/eurpub/ckac117)
Supplement: ckac117_Supplementary_Data [file ckac117_supplementary_data.zip › Appendix 2.docx]

**Appendix 2:** Supplementary evidence for Tables 1 and 4.

Sources for country information in Table 1:

| Country | Sources |
| --- | --- |
| Belgium | Taxation and Customs Union. [*Belgium: Droits d accise sur les eaux de boisson et les limonades/Accijnzen op alcoholvrije dranken 2015*](https://ec.europa.eu/taxation_customs/tedb/legacy/taxDetail.html?id=32/1424158940&taxType=Other+indirect+tax) [Accessed 20 November 2021].  University of North Carolina Chapel Hill (Global Food Research Program). [*Sugary drink taxes around the world*](https://globalfoodresearchprogram.org/wp-content/uploads/2020/08/SugaryDrink_tax_maps_2020_August_REV.pdf) [Updated August 2020, accessed 20 November 2021]. |
| Estonia | Köhler K, Reinap M. [Paving the way to a sugar-sweetened beverages tax in Estonia](https://apps.who.int/iris/handle/10665/325203). *Public Health Panorama*. 2017;3(4):537-820.  Vahtla A. [*Finance committee against adopting sweetened beverage tax unchanged*](https://news.err.ee/632560/finance-committee-against-adopting-sweetened-beverage-tax-unchanged). Eesti Rahvusringhääling. 6 September 2017 [Accessed 21 October 2021].  Knowledge4Policy (European Commission). [*Health Promotion and Disease Prevention Knowledge Gateway*](https://knowledge4policy.ec.europa.eu/health-promotion-knowledge-gateway/sugars-sweeteners-10_en) [Accessed 20 November 2021]*.* |
| Finland | University of North Carolina Chapel Hill (Global Food Research Program). [*Sugary drink taxes around the world*](https://globalfoodresearchprogram.org/wp-content/uploads/2020/08/SugaryDrink_tax_maps_2020_August_REV.pdf) [Updated August 2020, accessed 20 November 2021].  Knowledge4Policy (European Commission). [*Health Promotion and Disease Prevention Knowledge Gateway*](https://knowledge4policy.ec.europa.eu/health-promotion-knowledge-gateway/sugars-sweeteners-10_en) [Accessed 20 November 2021]*.*  Taxation and Customs Union. [*Finland: Virvoitusjuomavero/ Punktskatt på läskedrycker*](https://ec.europa.eu/taxation_customs/tedb/taxDetails.html?id=2001/1609455600) [Updated April 2021, accessed 20 November 2021].  Taxation and Customs Union. [*Finland:* Virvoitusjuomavero/ Punktskatt på läskedrycker](https://ec.europa.eu/taxation_customs/tedb/taxDetails.html?id=2001/1514764800) [Updated April 2018, accessed 20 November 2021].  Teivainen A. [*Finland to raise €75m by increasing alcohol and soft drink taxes in 2020*](https://www.helsinkitimes.fi/finland/finland-news/domestic/16552-finland-to-raise-75m-by-increasing-alcohol-and-soft-drink-taxes-in-2020.html)*.* Helsinki Times. 9 July 2019 [Accessed 20 November 2021].  Hagenaars LL, Jeurissen PPT, Klazinga NS. [The taxation of unhealthy energy-dense foods (EDFs) and sugar-sweetened beverages (SSBs): An overview of patterns observed in the policy content and policy context of 13 case studies](https://doi.org/10.1016/j.healthpol.2017.06.011). *Health Policy*. 2017;121(8):887-94. |
| France | University of North Carolina Chapel Hill (Global Food Research Program). [*Sugary drink taxes around the world*](https://globalfoodresearchprogram.org/wp-content/uploads/2020/08/SugaryDrink_tax_maps_2020_August_REV.pdf) [Updated August 2020, accessed 20 November 2021].  Hagenaars LL, Jeurissen PPT, Klazinga NS. [The taxation of unhealthy energy-dense foods (EDFs) and sugar-sweetened beverages (SSBs): An overview of patterns observed in the policy content and policy context of 13 case studies](https://doi.org/10.1016/j.healthpol.2017.06.011). *Health Policy*. 2017;121(8):887-94.  Le Bodo Y, Etilé F, Gagnon F, De Wals P. [Conditions influencing the adoption of a soda tax for public health: Analysis of the French case (2005–2012)](https://doi.org/10.1016/j.foodpol.2019.101765). *Food Policy*. 2019;88:101765.  République Française. [*Taxation des boissons 2021*](https://www.service-public.fr/professionnels-entreprises/vosdroits/F32101) [Accessed 20 November 2021]. |
| Hungary | Knowledge4Policy (European Commission). [*Health Promotion and Disease Prevention Knowledge Gateway*](https://knowledge4policy.ec.europa.eu/health-promotion-knowledge-gateway/sugars-sweeteners-10_en) [Accessed 20 November 2021]*.* |
| Kazakhstan | n/a |
| Latvia | University of North Carolina Chapel Hill (Global Food Research Program). [*Sugary drink taxes around the world*](https://globalfoodresearchprogram.org/wp-content/uploads/2020/08/SugaryDrink_tax_maps_2020_August_REV.pdf) [Updated August 2020, accessed 20 November 2021]. |
| Norway | Skattedirektoratet. [*Alkoholfrie Drinkevarer mv. 2020*](https://web.archive.org/web/20200930103411/https:/www.skatteetaten.no/globalassets/bedrift-og-organisasjon/avgifter/saravgifter/alkoholfrie-drikkevarer/2020-alkoholfrie-drikkevarer-ii-juli.pdf) [Accessed 20^th^ November 2021].  Kalle L-C. [*Slår full brus-alarm etter avgiftskutt*](https://www.nettavisen.no/okonomi/slar-full-brus-alarm-etter-avgiftskutt/s/12-95-3424139150?key=2021-06-13T08:49:42.000Z/retriever/41608d1c9bcf84a409bf747b30e4a25059f5bf13). Nettavisen Økonomi. 12 June 2021 [Accessed 1 November 2021]. |
| Portugal | University of North Carolina Chapel Hill (Global Food Research Program). [*Sugary drink taxes around the world*](https://globalfoodresearchprogram.org/wp-content/uploads/2020/08/SugaryDrink_tax_maps_2020_August_REV.pdf) [Updated August 2020, accessed 20 November 2021].  Goiana-da-Silva F, Cruz-e-Silva D, Gregório MJ, Miraldo M, Darzi A, Araújo F. [The future of the sweetened beverages tax in Portugal](https://doi.org/10.1016/S2468-2667(18)30240-8). *Lancet Pub Health*. 2018;3(12):e562.  Agence France-Presse. [*Portugal to levy sugar tax on soft drinks in 2017*](https://www.theguardian.com/society/2016/oct/15/portugal-to-levy-sugar-tax-on-soft-drinks-in-2017)*.* The Guardian. 15 October 2016 [Accessed 20 November 2021]. |
| Republic of Ireland | Irish Tax and Customs. [*Sugar Sweetened Drinks Tax (SSDT) 2021*](https://www.revenue.ie/en/companies-and-charities/excise-and-licences/sugar-sweetened-drinks-tax/index.aspx) [Accessed 20^th^ November 2021]. |
| United Kingdom | HM Revenue & Customs. [*Check if your drink is liable for the Soft Drinks Industry Levy*](https://www.gov.uk/guidance/check-if-your-drink-is-liable-for-the-soft-drinks-industry-levy) [Accessed 20^th^ November 2021]. |

Sources for evidential claims in Table 4:

| Argument | | Supported by independent evidence? | Details | Sources |
| --- | --- | --- | --- | --- |
| SSB taxation will harm the overall economy/ cost jobs | | No | Job losses in SSB-related sectors tend to be offset by job creation in others. The same applies to macroeconomic concerns. Moreover, this argument draws on an artificial trade-off between health and the economy. | Powell LM, Wada R, Persky JJ, Chaloupka FJ. [Employment impact of sugar-sweetened beverage taxes](https://doi.org/10.2105/AJPH.2013.301630). *Am J Public Health*. 2014;104(4):672-7.  Ecorys. [*Food taxes and their impact on competitiveness in the agri-food sector, a study*](https://www.google.com/url?sa=t&rct=j&q=&esrc=s&source=web&cd=&cad=rja&uact=8&ved=2ahUKEwjKorT4uuT5AhXSoFwKHVqRA_gQFnoECAsQAQ&url=https%3A%2F%2Fec.europa.eu%2Fdocsroom%2Fdocuments%2F5827%2Fattachments%2F1%2Ftranslations%2Fen%2Frenditions%2Fnative&usg=AOvVaw20-RsoRAqA7CK2t-dx8PCg). Brussels: European Commission; 2014.  Guerrero-López CM, Molina M, Colchero MA. [Employment changes associated with the introduction of taxes on sugar-sweetened beverages and nonessential energy-dense food in Mexico](https://doi.org/10.1016/j.ypmed.2017.09.001). *Prev Med*. 2017;105:S43-S9. |
| SSB taxation will harm businesses | Big businesses | No | No peer-reviewed, independent study has shown that SSB taxes harm employment or the food sector. Taxation is intended to reduce consumption of target products. However, existing evidence is insufficient to determine causal a relationship between SSB taxes and sector growth or changes to employment. Impact on share values appears minimal. | Law C, Cornelsen L, Adams J, Pell D, Rutter H, White M, et al. [The impact of UK soft drinks industry levy on manufacturers’ domestic turnover](https://doi.org/10.1016/j.ehb.2020.100866). *Econ Hum Biol*. 2020;37(100866).  Law C, Cornelsen L, Adams J, Penney T, Rutter H, White M, et al. [An analysis of the stock market reaction to the announcements of the UK Soft Drinks Industry Levy](https://doi.org/10.1016/j.ehb.2019.100834). *Econ Hum Biol*. 2020:100834. |
|  | Small businesses | No |  |  |
| SSB taxation poses an excessive administrative burden | | Uncertain | Fiscal measures do present administrative costs to companies, but well-designed taxes can minimise this. | Powell LM, Chriqui JF, Khan T, Wada R, Chaloupka FJ. [Assessing the potential effectiveness of food and beverage taxes and subsidies for improving public health: a systematic review of prices, demand and body weight outcomes](https://doi.org/10.1111/obr.12002). *Obes Rev*. 2013;14(2):110-28.  WHO Regional Office for Europe. [*Using price policies to promote healthier diets*](https://www.euro.who.int/__data/assets/pdf_file/0008/273662/Using-price-policies-to-promote-healthier-diets.pdf). Copenhagen: WHO Regional Office for Europe; 2015.  World Health Organization. [*Fiscal policies for diet and the prevention of noncommunicable diseases*](https://www.who.int/publications/i/item/9789241511247). Geneva: World Health Organization; 2016. |
| SSB taxation will lead to illicit or cross-border SSB trade | | Uncertain | Although cross-border trade has been observed in some settings, it is primarily a concern for local jurisdictions and the overall evidence is mixed. | Cawley J, Thow AM, Wen K, Frisvold D. [The economics of taxes on sugar-sweetened beverages: A review of the effects on prices, sales, cross-border shopping, and consumption](https://doi.org/10.1146/annurev-nutr-082018-124603). *Annu Rev of Nutr*. 2019;39(1):317-38.  Sassi F, Belloni A, Capobianco C. [*The Role of Fiscal Policies in Health Promotion*](https://www.oecd-ilibrary.org/docserver/5k3twr94kvzx-en.pdf?expires=1661516594&id=id&accname=guest&checksum=F3CC1F7D23822BCD022E88D216E5CB77). OECD Health Working Papers No 66. 2013. |
| SSB taxation is unfair/discriminatory towards industry | | n/a | Predominantly value-based/ legal matter. | n/a |
| SSB taxation will disproportionately affect poorer people | | Partially | Food and beverage taxes do present proportionally higher costs to low-income groups as these tend to spend a higher proportion of their disposable income on food compared to high-income groups. However, lower-income groups are also likely to benefit most from the health impacts of SSB taxes. | Ng SW, Rivera JA, Popkin BM, Colchero MA. [Did high sugar-sweetened beverage purchasers respond differently to the excise tax on sugar-sweetened beverages in Mexico?](https://www.cambridge.org/core/journals/public-health-nutrition/article/did-high-sugarsweetened-beverage-purchasers-respond-differently-to-the-excise-tax-on-sugarsweetened-beverages-in-mexico/37DBC66A6F1E19F74942888814EB1EA3/share/dd839df24b88c3856faee57b8d28bf0827f6af5b) *Public Health Nutr*. 2019;22(4):750-6.  Colchero MA, Popkin BM, Rivera JA, Ng SW. [Beverage purchases from stores in Mexico under the excise tax on sugar sweetened beverages: observational study](https://doi.org/10.1136/bmj.h6704). *BMJ*. 2016;352:h6704.  Thow AM, Downs S, Jan S. [A systematic review of the effectiveness of food taxes and subsidies to improve diets: understanding the recent evidence](https://doi.org/10.1111/nure.12123). *Nutr Rev*. 2014;72(9):551-65. |
| SSB taxation impedes on people's freedom of choice / government is overstepping (''nanny state') | | n/a | This is predominantly value-based so there is no evidence as such. | n/a |
| SSB taxation is a first step towards excessive regulation of other products ('slippery slope') | | n/a | This is predominantly value-based so there is no evidence as such. | n/a |
| Government is acting improperly (i.e., bad intentions, bad process) | | n/a | This is predominantly value-based so there is no evidence as such. | n/a |
| SSB taxation will not work (i.e., not reduce consumption or obesity/ NCDs) | | No | Health taxes have an established economic rationale and three systematic reviews suggest that SSB taxes (>10-20% at point of purchase) effectively reduce SSB consumption. Although SSB taxes in isolation are not enough to ‘solve’ the obesity problem, studies modelling the potential long-term impact on obesity rates predict significant declines. | Teng AM, Jones AC, Mizdrak A, Signal L, Genc M, Wilson N. [Impact of sugar-sweetened beverage taxes on purchases and dietary intake: Systematic review and meta-analysis](https://doi.org/10.1111/obr.12868). *Obes Rev*. 2019;20(9):1187-204.  Backholer K, Sarink D, Beauchamp A, Keating C, Loh V, Ball K, et al. [The impact of a tax on sugar-sweetened beverages according to socio-economic position: a systematic review of the evidence](https://www.cambridge.org/core/journals/public-health-nutrition/article/impact-of-a-tax-on-sugarsweetened-beverages-according-to-socioeconomic-position-a-systematic-review-of-the-evidence/587CFDC392441741771A93E7F652E222/share/18e0b96ff86396e27ab445520b110d7b87bb5184). *Public Health Nutr*. 2016;19(17):3070-84.  Redondo M, Hernández-Aguado I, Lumbreras B. [The impact of the tax on sweetened beverages: a systematic review](https://doi.org/10.1093/ajcn/nqy135). *Am J Clin Nutr*. 2018;108(3):548-63.  Basto-Abreu A, Barrientos-Gutiérrez T, Vidaña-Pérez D, Colchero MA, Hernández FM, Hernández-Ávila M, et al. [Cost-Effectiveness of the sugar-sweetened beverage excise tax in Mexico](https://doi.org/10.1377/hlthaff.2018.05469). *Health Aff*. 2019;38(11):1824-31.  Barrientos-Gutierrez T, Zepeda-Tello R, Rodrigues ER, Colchero-Aragonés A, Rojas-Martínez R, Lazcano-Ponce E, et al. [Expected population weight and diabetes impact of the 1-peso-per-litre tax to sugar sweetened beverages in Mexico](https://doi.org/10.1371/journal.pone.0176336). *PLoS One*. 2017;12(5):e0176336.  Sánchez-Romero LM, Penko J, Coxson PG, Fernández A, Mason A, Moran AE, et al. [Projected impact of Mexico's sugar-sweetened beverage tax policy on diabetes and cardiovascular disease: A modeling study](https://doi.org/10.1371/journal.pmed.1002158). *PLoS Med*. 2016;13(11):e1002158. |
| SSBs/sugar are not the problem (other products/behaviours are) | | No | Excess consumption of sugar, in particular SSBs, is a major cause of obesity, diabetes, and cardiovascular disease. | World Health Organization. [*Guideline: sugars intake for adults and children*](https://www.who.int/publications/i/item/9789241549028). Geneva: World Health Organization; 2015.  Malik VS, Hu FB. [Sugar-sweetened beverages and cardiometabolic health: An update of the evidence](https://doi.org/10.3390/nu11081840). *Nutrients*. 2019;11(8):1840.  Neelakantan N, Park SH, Chen G-C, van Dam RM. [Sugar-sweetened beverage consumption, weight gain, and risk of type 2 diabetes and cardiovascular diseases in Asia: a systematic review](https://doi.org/10.1093/nutrit/nuab010). *Nutr Rev*. 2021. |
| SSB taxation is not evidence-based | | No* | See above. While a lot of the real-world evidence on the effects of SSB taxation has emerged in recent years, a significant amount of evidence linking SSB consumption to negative health outcomes, and modelling studies on the impacts of SSB taxation have been available for longer. | Malik VS, Popkin BM, Bray GA, Després J-P, Hu FB. [Sugar-sweetened beverages, obesity, type 2 diabetes mellitus, and cardiovascular disease risk](https://doi.org/10.1161/CIRCULATIONAHA.109.876185). *Circulation*. 2010;121(11):1356-64.  Imamura F, O'Connor L, Ye Z, Mursu J, Hayashino Y, Bhupathiraju SN, et al. [Consumption of sugar sweetened beverages, artificially sweetened beverages, and fruit juice and incidence of type 2 diabetes: systematic review, meta-analysis, and estimation of population attributable fraction](https://doi.org/10.1136/bmj.h3576). *BMJ*. 2015;351:h3576.  Lal A, Mantilla-Herrera AM, Veerman L, Backholer K, Sacks G, Moodie M, et al. [Modelled health benefits of a sugar-sweetened beverage tax across different socioeconomic groups in Australia: A cost-effectiveness and equity analysis](https://doi.org/10.1371/journal.pmed.1002326). *PLoS Med*. 2017;14(6):e1002326.  Sánchez-Romero LM, Penko J, Coxson PG, Fernández A, Mason A, Moran AE, et al. [Projected impact of Mexico's sugar-sweetened beverage tax policy on diabetes and cardiovascular disease: A modeling study](https://doi.org/10.1371/journal.pmed.1002158). *PLoS Med*. 2016;13(11):e1002158. |
| Industry is already addressing obesity/NCDs through voluntary efforts | | No | Voluntary sugar reformulation efforts have not been as successful as regulatory efforts. Reformulation prompted by tiered SSB taxes, on the other hand, has significantly reduced sugar content in SSBs. Meaningful voluntary action and tools such as education should be seen as complementary to taxation. | Knai C, Petticrew M, Durand MA, Eastmure E, James L, Mehrotra A, et al. [Has a public–private partnership resulted in action on healthier diets in England? An analysis of the Public Health Responsibility Deal food pledges](https://doi.org/10.1016/j.foodpol.2015.04.002). *Food Policy*. 2015;54:1-10.  Bandy LK, Scarborough P, Harrington RA, Rayner M, Jebb SA. [Reductions in sugar sales from soft drinks in the UK from 2015 to 2018](https://doi.org/10.1186/s12916-019-1477-4). *BMC Med*. 2020;18(1):20.  Scarborough P, Adhikari V, Harrington RA, Elhussein A, Briggs A, Rayner M, et al. [Impact of the announcement and implementation of the UK Soft Drinks Industry Levy on sugar content, price, product size and number of available soft drinks in the UK, 2015-19: A controlled interrupted time series analysis](https://doi.org/10.1371/journal.pmed.1003025). *PLoS Med*. 2020;17(2):e1003025. |
| SSB taxation will have negative public health consequences | | No | While poorly designed taxes do present a risk of prompting switching to less healthy products, well-designed SSB taxes are unlikely to have this effect. | Finkelstein EA, Zhen C, Bilger M, Nonnemaker J, Farooqui AM, Todd JE. [Implications of a sugar-sweetened beverage (SSB) tax when substitutions to non-beverage items are considered](https://doi.org/10.1016/j.jhealeco.2012.10.005). *J Health Econ*. 2013;32(1):219-39.  Zhen C, Finkelstein EA, Nonnemaker J, Karns S, Todd JE. [Predicting the effects of sugar-sweetened beverage taxes on food and beverage demand in a large demand system](https://doi.org/10.1093/ajae/aat049). *Am J Agric Econ*. 2014;96(1):1-25.  Fletcher J, Frisvold D, Tefft N. [Substitution patterns can limit the effects of sugar-sweetened beverage taxes on obesity](http://dx.doi.org/10.5888/pcd10.120195). *Prev Chronic* *Dis*. 2013;10:E18-E. |
