# Supplementary material for: Corporate political activity in the context of sugar-sweetened beverage tax policy in the WHO European Region
Source: Eur J Public Health. 2022 Sep 13;32(5):786–93. doi: 10.1093/eurpub/ckac117 (PMC9527967; doi:10.1093/eurpub/ckac117)
Supplement: ckac117_Supplementary_Data [file ckac117_supplementary_data.zip › Appendix 3.docx]

**Appendix 3:** Most active opponents in SSB tax debates, based on respondents’ observations. The numbers refer to the number of participants who rated each group on the provided scale from *not involved* to *very active*.

| **Actor group** | **Not involved** | **Barely involved** | **Occasionally active** | **Regularly active** | **Very active** | **I don’t know** |
| --- | --- | --- | --- | --- | --- | --- |
| Large food/ beverage companies | - | - | 1 | 8 | 12 | 2 |
| Business associations | - | 1 | 3 | 4 | 11 | 4 |
| Small food/ beverage companies | 1 | 4 | 6 | 4 | 2 | 6 |
| Large retailers | 5 | 2 | 4 | 3 | 2 | 7 |
| Small retailers | 5 | 5 | 5 | - | - | 8 |
| Other (non-food/ beverage) companies | 4 | 5 | 3 | 1 | - | 10 |
| Politicians | 2 | 2 | 7 | 4 | 5 | 3 |
| Consumers or consumer groups | 8 | 6 | 5 | 4 | - | 3 |
| Charities/ NGOs | 7 | 4 | 3 | 1 | 2 | 6 |
| Think tanks/ research organisations | 7 | 4 | 4 | 3 | 1 | 3 |
| Individual academics/ researchers | 3 | 5 | 8 | 5 | - | 2 |
