# Supplementary material for: Corporate political activity in the context of sugar-sweetened beverage tax policy in the WHO European Region
Source: Eur J Public Health. 2022 Sep 13;32(5):786–93. doi: 10.1093/eurpub/ckac117 (PMC9527967; doi:10.1093/eurpub/ckac117)
Supplement: ckac117_Supplementary_Data [file ckac117_supplementary_data.zip › Appendix 4.docx]

**Appendix 4:** Active business associations and membership of leading SSB corporations. Searches for member companies were conducted in October 2021 and may thus not represent membership at the time when SSB taxes were under consideration in each country. Subsidiaries of multinational corporations are included.

| Country | Business association(s) | Member companies (2020 global market leaders in soft drinks) | |
| --- | --- | --- | --- |
|  |  | The Coca-Cola Company (& subsidiaries) | PepsiCo (& subsidiaries) |
| BE | [FIEB-VIWF (Belgian Federation of Bottled Water and Soft Drink Producers)](https://www.fieb-viwf.be/fr/la-fieb/membres/) | Yes | Yes |
| EE | Karastusjookide Tootjate Liit (Estonian Soft Drinks Association) | No website identified. Listed [contact email addresses belong to Coca-Cola Hellenic](https://www.inforegister.ee/en/80213365-KARASTUSJOOKIDE-TOOTJATE-LIIT-MTU). | |
|  | [Eesti Toiduainetööstuse Liit (Estonian Food Industry Association, member of Estonian Chamber of Commerce and Industry)](https://toiduliit.ee/liikmed/liikmete-nimekiri) | Yes | No |
|  | [Estonian Chamber of Commerce and Industry](https://www.koda.ee/en/members) | Yes | Yes |
|  | [Eesti Põllumajandus-Kaubanduskoja (Estonian Chamber of Agriculture and Commerce)](https://epkk.ee/wp-content/uploads/2021/10/2021-10-25_Members_of_ECAC.pdf) | No | No |
| FR | [Association Nationale de l’Industrie Alimentaire](https://www.ania.net/presentation-ania/organisation/conseil-dadministration) | No membership list identified, but Coca-Cola is represented in executive role | Not identifiable. |
|  | [Cultures Sucre](https://www.cultures-sucre.com/qui-sommes-nous/) | No | No |
|  | [Boissons Rafraîchissantes de France (member of ANIA)](https://www.boissonsrafraichissantes.com/) | Yes | Yes |
| HU | [Association of Responsible Food Producers (FÉSZ)](http://elelmiszeripar.hu/tagok/) | Yes | No |
|  | [Hungarian Mineral Water, Fruit Juice and Soft Drink Association (MAGYÜSZ)](https://italszovetseg.hu/tagjaink/) | Yes | No |
| IE | [Food Drink Ireland (Ibec trade association)](https://www.ibec.ie/connect-and-learn/industries/food-and-drink/food-drink-ireland) | No membership list identified | |
|  | [Irish Beverage Council (Ibec trade association)](https://www.ibec.ie/connect-and-learn/industries/food-and-drink/food-drink-ireland/irish-beverage-council) | No membership list identified | |
|  | [Restaurants Association of Ireland](https://www.rai.ie/about-rai/) | No membership list identified | |
| NO | [NHO Mat og Drikke (FoodDrinkNorway)](https://www.nhomd.no/dette-er-nho-mat-og-drikke/utvalg/) | Yes | Yes |
|  | [Virke (Federation of Norwegian Enterprise)](https://www.virke.no/english/) | No membership list identified | |
|  | [Bryggeri- og drikkevareforeningen (Norwegian Beer and Soft Drink Producers)](https://bryggeriforeningen.no/medlemsskap/vare-medlemmer/) | Yes | No |
| PO | [PROBEB (Portuguese association of refreshing non-alcoholic beverages)](https://www.probeb.pt/associados/) | Yes | Yes |
| KZ | No business associations identified |  |  |
| UK | [British Soft Drinks Association](https://www.britishsoftdrinks.com/Membership-Directory) | Yes | Yes |
|  | [Food and Drink Federation](https://www.fdf.org.uk/fdf/about-fdf/members-list/?SearchTerm=&Tag=&SortOrder=&PageSize=50&Show=) | Yes | Yes |
|  | [National Farmers Union](https://www.nfuonline.com/about-us/) | No membership list identified | |

**EU level business associations** which were identified as active in study countries:

- [UNESDA (Union of European Soft Drink Associations)](https://www.unesda.eu/our-members/)
  - In addition to Coca-Cola and PepsiCo, the following National business associations listed above are members:
    - Karastusjookide Tootjate Liit
    - Boissons Rafraîchissantes de France
    - Irish Beverage Council
    - PROBEB
    - FIEB VIWF
    - BSDA
- [FoodDrinkEurope](https://www.fooddrinkeurope.eu/members/)
  - In addition to Coca-Cola and PepsiCo, the following National business associations listed above are members:
    - NHO Mat og Drikke (NO)
    - FELELŐS ÉLELMISZERGYÁRTÓK SZÖVETSÉGE (HU)
    - Food and Drink Federation (UK)
    - Food Drink Ireland (IE)
    - ASSOCIATION NATIONALE DES INDUSTRIES ALIMENTAIRES (FR)
    - UNESDA (EU level)
- [Energy Drinks Europe](https://www.energydrinkseurope.org/about-ede/members/)
